# Association between role conflict and ambiguity and stress among nurses in primary health care centers in Saudi Arabia during the coronavirus disease 2019 pandemic: A cross-sectional study

**DOI:** 10.1097/MD.0000000000027294

**Published:** 2021-09-17

**Authors:** Saad A. Alyahya, Khalid A. Al-Mansour, Mohammed A. Alkohaiz, Mansour A. Almalki

**Affiliations:** a Nudge Unite, Innovation Center; b General Administration for Primary Health Centers, Ministry of Health; c Department of Social Studies, College of Arts, King Saud University, Riyadh; d Department of Sociology and Social Work, Facility of Arts and Humanities, King Abdulaziz University, Jeddah, Saudi Arabia.

**Keywords:** COVID-19, nurses, role ambiguity, role conflict, Saudi Arabia, stress

## Abstract

This study aimed to assess the association between role conflict and ambiguity among nurses in primary healthcare centers (PHCs) in Saudi Arabia and their stress levels during the coronavirus disease 2019 (COVID-19) pandemic.

In this online cross-sectional study, sociodemographic and occupational characteristics, role conflict, and ambiguity of 432 nurses were assessed using the Bowling Scale for Role Conflict and Ambiguity and stress was assessed using the 10-item Perceived Stress Scale from September 27 to October 17, 2020. Logistic regression was used to calculate odds ratios (ORs) and 95% confidence intervals (CIs) for above-median stress levels of nurses with average and high (2nd and 3rd tertiles) role conflict and ambiguity compared with nurses with low role conflict and ambiguity (1st tertile).

The mean (standard deviation) age of the nurses was 36.5 ± 6.6 years, and 25.9% of them were males. After adjusting for PHC type and working hours, nurses with average and high role conflict had significantly higher stress rates than those with low role conflict, with ORs (95% CIs) of 2.69 (1.62–4.46) and 6.31 (3.78–10.53), respectively. Similarly, nurses with average- and high-role ambiguity had significantly higher stress than those with low role ambiguity, with ORs (95% CIs) of 2.15 (1.30–3.55) and 7.68 (4.54–13.01), respectively. Increasing stress rates were detected across increasing categories of role conflict and ambiguity (*P* values for trend <.001).

We found that role conflict and ambiguity were associated with stress among nurses in PHCs in Saudi Arabia during the COVID-19 pandemic.

## 1 Introduction

In December 2019, a novel coronavirus, severe acute respiratory syndrome coronavirus 2, was identified in Hubei Province, China, and it spread across the country, resulting in the epidemic of coronavirus disease 2019 (COVID-19). After spreading to >110 countries, in March 2020, the World Health Organization (WHO) declared COVID-19 a pandemic.^[1]^ Within <9 months of this declaration, the WHO reported that approximately 1.5 million people worldwide lost their lives after being infected with the virus.^[2]^ The increased human loss was accompanied by a heavy financial bill attributed to lockdown measures, closing businesses, and suspending travel. Together, these factors have resulted in global stress and uncertainty regarding the future among the public worldwide.^[3–7]^

Nurses on the frontlines providing healthcare to patients with COVID-19 are exposed to more stressors than the public. They are also at a serious risk of contracting COVID-19 and possibly transmitting the virus to their colleagues, family, and friends. Furthermore, nurses experience increased workload with long hours and have additional shifts to meet the increasing number of patients and compensate for the shortages in medical staff attributed to the absence of infected nurses who had to isolate themselves.^[8–11]^ Moreover, there is a persistent need to provide sufficient personal protective equipment to general practitioners and clear guidance on patient care.^[12]^ All of these have resulted in increasing stress levels among nurses, which could likely increase errors and worsen medical outcomes.^[13–15]^

In Saudi Arabia, as a response to the increasing numbers of COVID-19 cases, the government imposed a state of lockdown, implemented measures to guarantee social distancing, closed schools and universities, suspended public transport, and prevented mass religious gatherings.^[16,17]^ In addition to these strict measures, it prioritized the early diagnosis and treatment of patients with COVID-19. To enhance capacity, the Saudi government prepared primary healthcare centers (PHCs) with trained nurses, diagnostic equipment, and radiological apparatus, allowing them to accept people with COVID-19 symptoms at any time with no previous appointment. In addition, >200 PHCs, named fever clinics, were devoted to receiving only patients with suspected COVID-19.^[18]^

One of the major challenges faced by nurses on the frontlines of Saudi PHCs were discrepancies in guidelines and protocols adopted to manage patients with COVID-19 along with continuous administrative and organizational changes to adapt to these modifications in guidelines and protocols. These discrepancies could lead to the absence of precise job descriptions that could result in role conflict and ambiguity.^[19,20]^ Although role conflict reflects inconsistent or incompatible sets of expectations and demands at work, role ambiguity reflects uncertainty and information deficit concerning which tasks and responsibilities individuals should undertake.^[21,22]^ Role conflict and ambiguity among health care workers have been shown to result in work stress and deteriorated health care quality.^[23–25]^

However, the possible association between role conflict and ambiguity and the risk of stress among nurses working at PHCs in Saudi Arabia during the COVID-19 pandemic has not been investigated. Because nurses on the frontlines in Saudi Arabia experience enormous stress levels related to the COVID-19 pandemic, role conflict and ambiguity could theoretically augment this stress. Given the fact that the performance of nurses is vital to quality patient care, this study aimed to investigate the association between role conflict and ambiguity among nurses in PHCs in Saudi Arabia and assess their stress levels during the COVID-19 pandemic as part of a larger project assessing work-related challenges affecting health care workers in the country.^[26]^ We believe that the findings of this study may help provide better knowledge of stressors affecting nurses during the COVID-19 pandemic.

## 2 Methods

### 2.1 Study design, study population, and settings

A multi-stage random sampling approach was used to recruit nurses working at different PHCs in Saudi Arabia to participate in this cross-sectional study. First, Saudi Arabia was divided into 5 geographic regions: eastern, western, northern, southern, and central. The Saudi Minister of Health is represented in the 5 regions by 22 Directorates of Health Affairs that supervise hundreds of PHCs across the country. In each directorate, 8 PHCs were randomly selected by card withdrawal and based on eligibility criteria. All nurses working at the selected PHCs received e-mails inviting them to participate in the study. The heads of the selected PHCs were informed of the study and were instructed to encourage nurses to respond; however, no rewards were offered. Nurses who agreed to participate received another e-mail with an online questionnaire assessing their role conflict and ambiguity, perceived stress, and other sociodemographic and occupational data. Data collection started on September 27, 2020, and nurses were given 3 weeks to respond. Reminders were sent on October 4, 2020. The questionnaire included three sections: section I assessed sociodemographic and occupational characteristics of nurses, section II assessed their role conflict and ambiguity, and section III assessed their perceived stress. The anticipated time to complete the questionnaire was approximately 10 minutes. The eligibility criteria were as follows: nurses, presently working at PHCs in Saudi Arabia, and aged ≥18 years. Subsequently, 432 nurses responded to the questionnaire, with a response rate of 68%. The study hypothesis is that role conflict and role ambiguity predict stress levels among nurses in PHCs.

### 2.2 Exposures

Role conflict and ambiguity among nurses were assessed using the Bowling Scale for Role Conflict and Ambiguity. This scale is composed of 12 statements: 6 for role conflict and 6 for role ambiguity. Role conflict included the following statements: “In my job, I often feel like different people are pulling me in different directions,” “I have to deal with competing demands at work,” “My superiors often tell me to do 2 different things that cannot both be done,” “The tasks I am assigned at work rarely come into conflict with each other,” “The things I am told to do at work do not conflict with each other,” and “In my job, I’m seldom placed in a situation where one job duty conflicts with other job duties.” Role ambiguity included the following statements: “I am not sure what is expected of me at work,” “The requirements of my job aren’t always clear,” “I often don’t know what is expected of me at work," “I know everything that I am expected to do at work with certainty, “My job duties are clearly defined,” and “I know what I am required to do for every aspect of my job.” Respondents were instructed to express how much they agreed with these statements on a Likert scale from one (strongly disagree) to seven (strongly agree), with higher scores indicating higher levels of role conflict and ambiguity. The last 3 statements in the Role Conflict Scale and Role Ambiguity Scale were provided reverse scores. This scale, upon examination of 5 different datasets, displayed high levels of validity, internal consistency, and test-retest reliability.^[27]^

### 2.3 Outcomes

We used the 10-item Perceived Stress Scale (PSS-10) to assess stress among nurses. PSS-10 is a global measure of perceived stress, and respondents express the frequency of exposure to nonspecific stressors during the previous month. Participants responded to these stressors using a Likert scale that ranged from 0 (never) to 4 (very often), with higher scores indicating higher levels of stress. Four items were provided reversed scores. The scale showed high validity and reliability in previous studies.^[28]^

### 2.4 Covariates

Our online survey included other variables that were included in the regression models: age in years, sex (male or female), marital status (married or unmarried), smoking status (present smoker or nonsmoker), educational level (university degree or high school and equal degrees), working hours per week, and PHC type (regular PHC or fever clinics specialized in managing people with COVID-19 symptoms).

### 2.5 Ethical considerations

This study was reviewed and approved by the Central Institutional Review Board of the Saudi Ministry of Health (approval number: 21-37 M, date of approval: March 25, 2021). The study was conducted in accordance with the principles of the Declaration of Helsinki. Informed consent was obtained from all the participants involved in this study.

### 2.6 Statistical analyses

Since there are no agreed-on cut-offs for the Role Conflict Scale and Role Ambiguity Scale, the highest tertile (3rd) was defined as having high role conflict and ambiguity, the middle tertile (2nd) was defined as having average role conflict and ambiguity, and the lowest tertile (1st) was defined as having low role conflict and ambiguity. In contrast, nurses with a PSS-10 score higher than the median score were considered to have higher stress levels than their counterparts with below-median scores.

Descriptive statistics of the participating nurses divided by their role conflict and ambiguity were determined, and *χ*^2^ and 1-way analysis of variance tests were used to calculate *P* values that assessed sociodemographic and occupational differences among the groups. Logistic regression analyses were used to compute odds ratios (ORs) and their 95% confidence intervals (CIs) for above-median stress levels of nurses with high and average role conflict and ambiguity compared with nurses with low role conflict and ambiguity. Two models of regression analyses were used: unadjusted and adjusted for working hours and PHC type, respectively. Data were analyzed using the Statistical Package for the Social Sciences (SPSS, version 25, Chicago, IL).

## 3 Results

Among 432 included nurses with a mean (standard deviation) age of 36.5 ± 6.6 years, 25.9% were male, 80.3% were married, 21.3% held university or higher degrees, 12.5% were present smokers, and 78.7% were working at regular PHCs; the mean (standard deviation) working hours were 42.9 ± 8.0 hours per week. The cutoff values for role conflict scale according to the tertiles were as follows: 6 to 21 for low, 22 to 27 for average, and 28 to 42 for high role conflict, whereas the opposite cut-values in role ambiguity were 6 to 13 for low, 14 to 22 for average, and 23 to 42 for high role ambiguity. Apart from the type of PHC, which differed significantly across role ambiguity categories, all other sociodemographic and occupational characteristics did not show statistically significant differences among nurses according to their role conflict or ambiguity category (Table 1).

**Table 1:** Characteristics of nurses in Saudi Arabia distributed by their role conflict and ambiguity.

		Role conflict				Role ambiguity			
Characteristics	Overall	Low(6–21)	Average(22–27)	High(28–42)	*P*	Low(6–13)	Average(14–22)	High(23–42)	*P*
No.	432	155 (35.9)	137 (31.7)	140 (32.4)	—	158 (36.6)	140 (32.4)	134 (31.0)	—
Age, y (mean ± SD)	36.5 ± 6.6	36.6 ± 7.1	36.8 ± 6.9	35.9 ± 5.7	.503	37.1 ± 6.9	35.8 ± 5.9	36.4 ± 6.9	.228
Sex									
Male	112 (25.9)	39 (25.2)	36 (26.3)	37 (26.4)	.963	38 (24.1)	44 (31.4)	30 (22.4)	.185
Female	320 (74.1)	116 (74.8)	101 (73.7)	103 (73.6)		120 (75.9)	96 (68.6)	104 (77.6)	
Marital status									
Married	347 (80.3)	126 (81.3)	109 (79.6)	112 (80.0)	.927	126 (79.7)	116 (82.9)	105 (78.4)	.628
Others	85 (19.7)	29 (18.7)	28 (20.4)	28 (20.0)		32 (20.3)	24 (17.1)	29 (21.6)	
Educational level									
University	92 (21.3)	30 (19.4)	30 (21.9)	32 (22.9)	.748	35 (22.2)	29 (20.7)	28 (20.9)	.946
High school	340 (78.7)	125 (80.6)	107 (78.1)	108 (77.1)		123 (77.8)	111 (79.3)	106 (79.1)	
Smoking status									
Current	54 (12.5)	23 (14.8)	13 (9.5)	18 (12.9)	.382	20 (12.7)	17 (12.1)	17 (12.7)	.988
Non-current	378 (87.5)	132 (85.2)	124 (90.5)	122 (87.1)		138 (87.3)	123 (87.9)	117 (87.3)	
Health center									
Regular	340 (78.7)	124 (80.0)	106 (77.4)	110 (78.6)	.860	136 (86.1)	107 (76.4)	97 (72.4)	.013
Fever	92 (21.3)	31 (20.0)	31 (22.6)	30 (21.4)		22 (13.9)	33 (23.6)	37 (27.6)	
Working h/wk (mean ± SD)	42.9 ± 8.0	41.9 ± 5.9	43.8 ± 8.5	43.1 ± 9.3	.126	42.2 ± 4.6	43.4 ± 9.2	43.2 ± 9.6	.356

SD = standard deviation.

Nurses with average and high role conflict had significantly higher levels of stress than those with low role conflict in the unadjusted model, with ORs (95% confidence intervals [CIs]) of 2.73 (1.65–4.52) and 6.34 (3.80–10.56), respectively. These associations did not change after controlling for PHC type and working hours (2.69 [1.62–4.46] and 6.31 [3.78, 10.53], respectively). Similarly, nurses with average and high role ambiguity had significantly higher levels of stress than those with low role ambiguity in the unadjusted model, with ORs (95% CIs) of 2.17 (1.32–3.56) and 7.69 (4.57–12.96), respectively. These associations did not change after controlling for PHC type and working hours (2.15 [1.30–3.55] and 7.68 [4.54–13.01], respectively). Significant trends were observed in both models across increasing role conflict and role ambiguity (all *P* values for trend <.001) (Table 2).

**Table 2:** The association between role conflict and ambiguity of nurses in Saudi Arabia and their stress levels.

Variables	PSS-10 > 20n (%)	Model IOR (95% CI)	Model IIOR (95% CI)
Role conflict			
Low	36 (23.2)	1	1
Average	62 (45.3)	2.73 (1.65–4.52)	2.69 (1.62–4.46)
High	92 (65.7)	6.34 (3.80–10.56)	6.31 (3.78–10.53)
*P* value for trend		<.001	<.001
Role ambiguity			
Low	38 (24.1)	1	1
Average	57 (40.7)	2.17 (1.32–3.56)	2.15 (1.30–3.55)
High	95 (70.9)	7.69 (4.57–12.96)	7.68 (4.54–13.01)
*P* value for trend		<.001	<.001

CIs = confidence intervals, ORs = odds ratios, PSS-10 = 10-item Perceived Stress Scale. Model I: Unadjusted. Model II: Adjusted for working hours and primary healthcare center type.

## 4 Discussion

This study indicated that during the COVID-19 pandemic, nurses working in PHCs in Saudi Arabia and having average and high role conflict and ambiguity were significantly more likely to have higher levels of stress than their counterparts with low role conflict and ambiguity. This result is not surprising because little is known about the COVID-19 pandemic, which has led to continuous changes in COVID-19 policies, which is expected to create role conflict and ambiguity because it is difficult to adapt to rapid changes that affect nurses’ roles. A dose–response relationship was detected with higher role conflict, and ambiguity levels were associated with higher stress levels.

Our results are consistent with those of previous studies conducted before and during the COVID-19 pandemic. In one study that included 146 nurses from 3 large hospitals in India, role conflict and ambiguity were associated with work stress.^[23]^ Another study conducted among 170 physicians and 81 nurses from one university hospital in Turkey showed a strong positive correlation between role conflict and ambiguity and work stress, and role conflict and ambiguity could explain higher levels of work stress reported by nurses than by physicians.^[29]^ In a study on primary healthcare workers who worked on the frontline in Saudi Arabia during the COVID-19 pandemic, both work ambiguity and conflict were significant predictors of stress.^[30]^

Stress among nurses can have adverse effects on patient outcomes. In a cross-sectional survey of 820 nurses and 621 patients, stress levels among nurses were inversely correlated with patient satisfaction.^[31]^ Another cross-sectional study of linked data between 10,184 nurses and 232,342 patients revealed that work stress was related to failure to rescue and even increased mortality.^[32]^

Notably, stress is not the only consequence of role conflict and ambiguity in health care settings; a previous study showed that they can undermine healthcare quality. One study conducted 82 interviews with randomly selected healthcare workers in a Swiss hospital and concluded the potential effects of role conflict and ambiguity on the quality of patient care with significant failure to provide efficient, patient-centered, and timely health care.^[25]^

It should be noted that the strength of this study was that it was the first study to investigate the association between role conflict and ambiguity and stress among nurses during the COVID-19 pandemic using a multi-stage random sampling approach to recruit a representative sample of nurses and using validated data collection tools to assess exposures and outcomes. However, this study had some limitations. First, the cross-sectional design of this study does not imply causality. A prospective design to assess the temporal association between role conflict and ambiguity and the risk of stress is recommended in future studies. Second, online surveys are vulnerable to many types of selection bias, and this study is no exception.^[33]^ Third, the relatively low response rate might hide nonresponse bias because we cannot guarantee that nurses who did not respond to the questionnaire had the same sociodemographic and occupational characteristics as those who responded.

In conclusion, this study demonstrated that role conflict and ambiguity among nurses managing patients with COVID-19 in PHCs in Saudi Arabia were closely associated with their stress levels. All organizational and structural changes that have been implemented in PHCs in Saudi Arabia during the COVID-19 pandemic should be reviewed to identify possible sources of role conflict and ambiguity to avoid work stress among nurses. The policies can be used to increase nurses’ roles and responsibilities in PHCs, which might ease adaptation to new challenges. This can help reduce stress levels among nurses, thereby helping protect the quality of patient care. Continuous evaluation of role conflict and ambiguity needs to be implemented to detect any problems or difficulties that might affect stress levels of nurses in PHCs, which subsequently affects the quality of health care services provided. Further studies are needed to elucidate other possible effects of role conflict and ambiguity on the quality of health care and outcomes of COVID-19 management.

## Acknowledgments

The authors thank the Director General of the General Administration of Health Centers Affairs at the Saudi Ministry of Health. Moreover, we would also like to thank everyone who worked on the data collection team, and finally, we extend our gratitude to all the participants and health practitioners in this study.

## Author contributions

**Conceptualization:** Saad A. Alyahya, Mohammed A. Alkohaiz, Mansour A. Almalki.

**Data curation:** Khalid A. Al-Mansour.

**Formal analysis:** Saad A. Alyahya, Mansour A. Almalki.

**Investigation:** Khalid A. Al-Mansour, Mansour A. Almalki.

**Methodology:** Saad A. Alyahya, Mohammed A. Alkohaiz, Mansour A. Almalki.

**Project administration:** Saad A. Alyahya.

**Resources:** Khalid A. Al-Mansour, Mohammed A. Alkohaiz, Mansour A. Almalki.

**Software:** Saad A. Alyahya, Mohammed A. Alkohaiz.

**Supervision:** Khalid A. Al-Mansour, Mohammed A. Alkohaiz.

**Validation:** Khalid A. Al-Mansour.

**Visualization:** Mansour A. Almalki.

**Writing – original draft:** Saad A. Alyahya, Khalid A. Al-Mansour, Mohammed A. Alkohaiz, Mansour A. Almalki.

**Writing – review & editing:** Saad A. Alyahya, Khalid A. Al-Mansour, Mohammed A. Alkohaiz, Mansour A. Almalki.
